# A Comparison of Digestive Strategies for *Teratoscincus roborowskii* With Different Diet Compositions: Digestive Enzyme Activities, Gut Microbiota, and Metabolites

**DOI:** 10.1002/ece3.70751

**Published:** 2024-12-22

**Authors:** Ziyi Wang, Ruichen Wu, Yi Yang

**Affiliations:** ^1^ Xinjiang Key Laboratory for Ecological Adaptation and Evolution of Extreme Environment Biology, College of Life Sciences Xinjiang Agricultural University Urumqi China

**Keywords:** dietary intervention, digestive enzyme, gut microbiota, metabolomics, *Teratoscincus roborowskii*

## Abstract

Animal gut microbiota play important roles in host immunity, nutrient metabolism, and energy acquisition. The gut microbiota and its metabolic products interact with the host in many different ways, influencing gut homoeostasis and health. *Teratoscincus roborowskii* is an endemic species which displays special frugivorous behavior, and it has been observed consuming grapes. To explore the effects of grape intake on the gut microbiota and metabolites of *T. roborowskii* , 16S rRNA sequencing and liquid chromatography mass spectrometry metabolomics were applied to investigate the gut microbiota and metabolite profiles of *T. roborowskii* fed with mealworms (LC group) and a mixture of mealworms and grapes (FG group). Our results demonstrated that a notable shift in microbiota composition occurred, particularly in terms of an increase in the probiotic *Lactococcus* in the FG group. The metabolite analysis revealed a significant enrichment of the pathways related to glucose metabolism in the FG group. In addition, the digestive enzyme activity analysis showed that the α‐amylase and cellulase activities in the FG group were significantly higher than those of the LC group, which was consistent with the food type. A strong correlation between diet, gut microbiota, and fecal metabolites was observed, which may imply that different diets promote the establishment of host intestinal adaptation strategies. Our study provides a theoretical basis for host health and the scientific captive breeding of the desert lizards *T. roborowskii* .

## Graphical abstract

*Teratoscincus roborowskii* is an endemic species which displays special frugivorous behavior, and it has been observed consuming grapes. To explore the effects of grape intake on the gut microbiota and metabolites of *T. roborowskii* , 16S rRNA sequencing and liquid chromatography mass spectrometry metabolomics were applied to investigate the gut microbiota and metabolite profiles of *T. roborowskii* fed with a mixture of mealworms and grapes. Our results demonstrated that a notable shift in microbiota composition occurred after the addition of grapes to their diet, particularly in terms of an increase in the probiotic Lactococcus. The metabolite analysis revealed a significant enrichment of the pathways related to glucose metabolism. And the α‐amylase and cellulase activities were significantly higher. A strong correlation between diet, gut microbiota, and fecal metabolites was observed, which may imply that different diets promote the establishment of host intestinal adaptation strategies.

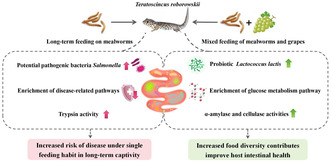

## 1 Introduction

Animal intestines have diverse, and dynamically changing bacterial communities that play important roles in host immunity, nutrient metabolism, and energy acquisition (Ding et al. 2017; Visconti et al. 2019). A number of factors have been either correlated with microbiome diversity in the animal intestine or have been experimentally shown to modulate it, such as captivity, dietary changes, seasonal variation, environmental factors, and enteric pathogens (Johnson et al. 2019; Price et al. 2017; Jiang et al. 2017; Amato et al. 2013); the consistently dominant drivers appear to be host evolutionary history and diet (Youngblut et al. 2019). Diet is thought to have a strong and pervasive influence on gut microbiota composition across vertebrates (Kartzinel et al. 2019). The gut microbiota has a great degree of plasticity and a rapid response ability, and it can undergo rapid and significant changes in response to short‐term diet interventions. For example, after a short‐term (6 days) feeding of hybrid groupers with different lipid‐levels diets, the composition, diversity, and functional pathways of the intestinal microbes in the fish were significantly changed, and different lipid metabolism patterns were produced (Xu et al. 2022). Captive *Varanus salvator* fed with eggs, bullfrogs, or depilated chickens showed different dominant gut bacteria in each diet (Du et al. 2022). In addition, feeding habits can also affect the gut microbiota of *Shinisaurus crocodilurus* , with potential effects on host health due to the influence of diet on the abundances of pathogenic or opportunistic gut bacteria (Jiang et al. 2017).

A mutually beneficial relationship between the host and its resident microbiota results in the production of metabolites by microbes that contribute to the evolutionary fitness of the host (Nicholson et al. 2012). Metabolomics has emerged as a technique that focuses on defining the functional status of hostemicrobial relationships in biological specimens, such as urine, blood, feces, and tissues (Chen et al. 2019). Multiple bacterial genomes can sequentially modulate metabolic reactions, resulting in combinatorial metabolism of substrates by the microbiome and host genome, exemplified by production of bile acids, choline, and short‐chain fatty acids (SCFAs) that are essential for host health (Nicholson and Wilson 2003). In addition, the production of metabolites by microbes contributes to the host metabolic phenotype and hence to disease risk. The profound influence of the gut microbiota on the host immune system is strongly associated with long‐term health prospects (Nicholson et al. 2012). Therefore, the metabolomic analysis of the gut microbiota is an emerging tool for exploring the effects of the different influencing factors on animals for better conservation (Holmes et al. 2012). Detecting complex changes in metabolite levels can aid disease diagnosis and can also monitor cellular responses to nutrition, drugs, toxins, and environmental factors (Tran, McConville, and Loukopoulos 2020; Kim et al. 2016). Any differences in the microbial community may have a significant effect on the metabolite profiles of the host, which can be explored through untargeted metabolomics (Jo et al. 2021). The formation of microbial metabolites is strongly influenced by dietary intake, particularly that of nondigestible dietary carbohydrates, protein, and fat. This is dictated primarily by the chemical structures of the substrates themselves and the microbial pathways by which they are processed (Flint et al. 2015). It was shown that the intake of different dietary fiber produced different metabolic responses (An increase or decrease in fatty acids, amino acids, etc.) in mice colonized with different gut microbes (Murga‐Garrido et al. 2021).

The digestive enzyme activity of animals is an important index reflecting digestive physiological function, which can be roughly divided into protease, amylase, lipase, and cellulase according to the different objects of digestion (Xu 2006). Indeed, correlations between carbohydrase activities in the gut and carbohydrate intake in the natural diet have been observed in fishes, birds, and mammals, even when analyzed in a phylogenetic context (Wehrle et al. 2020). There should be a match between gut function (digestive enzyme activities and nutrient transport rates) and the food ingested by an animal (Karasov and Douglas 2013). Thus, to maximize net nutrient gain, a diet shift should lead to changes in gut physiology to match the new diet, over short or long timescales. For example, increased digestive substrate concentration (e.g., starch) requires increases in matched enzyme activities (e.g., amylase activity) to achieve high digestibility of the nutrient (Karasov and Martínez del Rio 2007). Beyond endogenous digestive processes, such as digestive enzyme synthesis and secretion, many herbivores and omnivores rely on microbial symbioses, usually in the hindgut, to digest more fibrous portions of plants (e.g., cellulose) and can derive some portion of their energy intake from microbial fermentation (McBee and McBee 1982; Bjorndal 1997). For example, studies on fish with different feeding habits (omnivorous, herbivorous, plankton feeder, and carnivorous) have shown that feeding habits determine the digestive enzyme activities and differential colonization of intestinal flora; the trypsin and amylase activities were significantly higher in the carnivorous and herbivorous fishes, respectively (Jiao et al. 2023).

**FIGURE 1:**
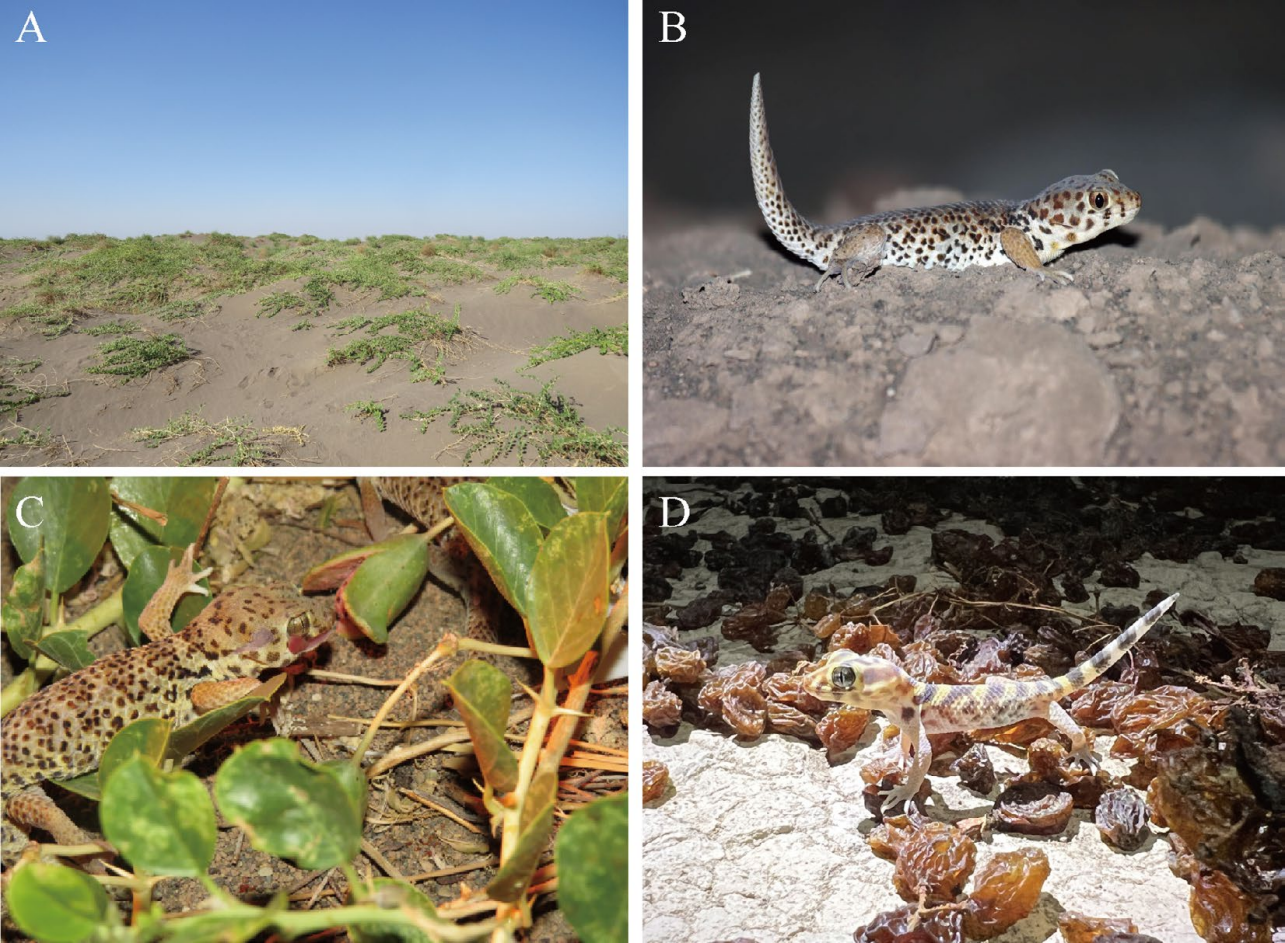
(A) Habitat of the *T. roborowskii* ; (B) Wild *T. roborowskii* ; (C) *T. roborowskii* feeding on *C. spinosa* fruit; (D) *T. roborowskii* active in the area where grapes are being dried.

*Teratoscincus roborowskii* is an endemic species that is only distributed in the Turpan Depression of the Xinjiang Uyghur Autonomous Region, China (Figure 1). The investigation of *T. roborowskii* has mainly focused on behavior, ecology, and morphology, encompassing mimicry (Autumn and Han 1989), foraging modes (Werner et al. 1997), activity rhythm (Song et al. 2017), sexual dimorphism, diet, skeletochronology (Li, Song, and Shi 2010), home range (Li, Song, and Shi 2013), habitat (Song et al. 2017), seed dispersal (Yang, Lin, and Shi 2021), and the digestive tract morphology (Wang et al. 2024). Many studies have reported that grapes exhibit a variety of biological activities, such as antioxidant, gut‐microbiota regulating, cardioprotective, antidiabetic, and anticancer activities (Zhou et al. 2022). The health benefits of grapes are largely attributed to their rich bioactive compounds, especially polyphenols. Among the beneficial effects of polyphenols in body health, special attention is currently paid to their capacity to modulate the composition and metabolic activity of gut microbiota, which is associated with an increase in probiotics and prebiotics (Zorraquín et al. 2020).

Field observation and dietary analysis have shown that the dietary habits of *T. roborowskii* display a significant seasonal shift. *T. roborowskii's* main food sources are insects in spring, whereas they eat a lot of *Capparis spinosa* fruits in summer and autumn, which lead to seasonal shifts in gut microbiota and metabolites. Verrucomicrobia and Proteobacteria exhibited dynamic ebb and flow patterns between spring and autumn. Metabolomic profiling also revealed differences mainly related to the formation of secondary bile acids (Gao, Yang, and Shi 2023). Recently, we have also observed that *T. roborowskii* has shown grape‐eating behavior. Currently, *T. roborowskii* is listed as a second‐class protected animal; nevertheless, few studies related to diet have been conducted in captivity. In traditional captivity, they were simply given free access to mealworms. Here, combining their behavior of feeding on grapes in the wild, we added grapes to its diet, and addressed the following questions: what effect does the addition of grapes to the diet have on the gut microbiota and metabolome of *T. roborowskii*? Could the addition of grapes to the diet increase the abundance of potential probiotics in the gut and confer health benefits to *T. roborowskii*? In the present study, we compared the lizards' and their gut microbiomes' responses to diets including or excluding grapes. We examined the lizards' digestive functionality via measurements of digestive enzyme activities, gut microbiome composition using 16s rRNA gene amplicon sequencing, and microbiome metabalome using Liquid chromatography mass spectrometry (LC–MS)‐based metabolomics. Understanding the changes in the microbiota and metabolites of *T. roborowskii* with different diets will provide a theoretical basis for the host health and the scientific captive breeding of desert lizards.

## 2 Materials and Methods

### 2.1 Animal and Feces Collection

*Teratoscincus roborowskii* individuals were captured in May 2021 at the Turpan Eremophytes Botanic Garden at the Chinese Academy of Sciences, which is located in the Turpan Basin in the Xinjiang Uyghur Autonomous Region of China (E89°11′, N42°54′). Five healthy adult lizards were selected because they had been kept in captivity for a long time in the same environment and had a uniform physiological state. Each individual was kept in captivity for up to 4 months in 30 × 21 × 15.5 cm (L × W × H) plastic rearing boxes at room temperature which were regularly cleaned. During captivity, the lizards were fed with three to four live mealworms and necessary trace elements every other day, such as vitamin water and calcium powder. Fecal samples were collected after 4 months. Observed the excretion of lizard feces every 3 h throughout the day to ensure that the collected feces were fresh, as long‐term captivity (LC) group samples. Afterwards, the *T. roborowskii* were subjected to a 14‐day fasting period to empty their digestive tracts of feces. Then, the lizards were fed with mealworms and peeled grapes, which were thoroughly mixed with a homogenizer at a ratio of 1:1. The nutrient composition of the two diets is shown in Table 1. The lizards were given free access to a sufficient amount of homogenized food; fresh fecal pellets were collected and were considered as feeding grapes (FG) group samples. All the fecal samples were collected into sterile cryovials using sterilized tweezers and were snap‐frozen in liquid nitrogen immediately; then, they were stored at −80°C. All the experimental procedures involving animals were approved by the Animal Welfare and Ethics Committee of Xinjiang Agricultural University, Urumqi, Xinjiang, China. After the experiment was completed, all the lizards were released into the Turpan Eremophytes Botanic Garden.

**TABLE 1:** Compostion of diets fed to *T. roborowskii* .

Nutritional composition	Diet (% dry mass)	
	Mealworms	Grape + mealworms
Dry matter	99.88	89.23
Organic matter	96.30	95.96
Fat	44.30	9.99
Coarse fiber	1.68	4.08
Crude protein	46.14	30.99
Ca	2.67	2.13
P	0.66	2.00
Energy (MJ/kg)	14.79	21.70

*Note:* Composition is based on percent dry matter.

### 2.2 Assessment of Digestive Enzyme Activity

A 0.02 M pH 7.5 phosphate buffer solution with disodium hydrogen phosphate and sodium dihydrogen phosphate was prepared. Then, 0.1 g of the homogenized fecal sample of the individual was placed into 1 mL of phosphate buffer solution and homogenized thoroughly. It was then placed in a refrigerated centrifuge and centrifuged for 20 min (0°C–4°C, 12,000 rpm). The supernatant obtained was the crude enzyme solution, which should be stored in an ultra‐low‐temperature refrigerator and measured as soon as possible. Each sample had three biological replicates.

#### 2.2.1 Assessment of Protein Concentration

The BCA protein concentration assay kit (Biosharp, Hefei, China; Cat. No. BL521A) was used for this assay. In alkaline environment, Cu^2+^ in the BCA reagent could be reduced to Cu^+^, and Cu^+^ reacted with the BCA reagent to form a blue‐purple complex with a strong absorption peak at 562 nm. Experimental procedures—bicinchoninic acid (BCA) solution was added to the crude enzyme solution, and the mixture was mixed in a water bath at 37°C for 30 min. The mixed solution was poured into cuvette and the absorbance value was measured at a wavelength of 562 nm using a spectrophotometer (722 N visible spectrophotometer, Jinghua instrument Inc. Shanghai, China). A standard curve was drawn using a bovine serum protein (BSA) standard solution to calculate the protein concentration in the crude enzyme solution. It was used for subsequent calculations of digestive enzyme activities.

#### 2.2.2 Assessment of Trypsin Activity

The trypsin assay kit (Nanjing Jiancheng, Nanjing, China; Cat. No. A080‐2) was used for this assay. Trypsin can catalyze the hydrolysis of the ester chain of the substrate arginine ethyl ester, leading to an increase in absorbance at a wavelength of 253 nm. The activity of the enzyme can be calculated based on the change in absorbance value. Experimental procedures: The colorimetric tank of the spectrophotometer was preheated at 37°C in advance. The timing was started while the crude enzyme solution obtained from the extraction was mixed with the substrate (ethyl arginine ester) and then poured into a cuvedish (0.5 cm optical path). Absorbance values were measured and recorded at 253 nm and again 20 min later. The change in absorbance value of 0.003 per minute caused by trypsin contained in each gram of protein is defined as one enzyme activity unit (U/mg prot).

#### 2.2.3 Assessment of Lipase Activity

The lipase assay kit (Nanjing Jiancheng, Nanjing, China; Cat. No. A054‐1‐1) was used for this assay. The triglycerides in the micelles undergo hydrolysis under the action of lipase, causing the micelles to break apart, resulting in a decrease in scattered light or turbidity. At a wavelength of 420 nm, turbidity is measured and the activity of lipase is calculated. Experimental procedures: The colorimetric tank of the spectrophotometer was preheated at 37°C in advance. The crude enzyme solution was mixed with the substrate solution (triacylglycerides) and poured into a cuvette, and its absorbance value was measured at a wavelength of 420 nm and again after 10 min. The change in absorbance value was substituted into the formula for calculation. One unit of enzyme activity (U/mg prot) is defined as the lipase in 1 g of protein consuming 1 μmol of substrate in 1 min.

#### 2.2.4 Assessment of α‐Amylase Activity

The α‐amylase assay kit (Nanjing Jiancheng, Nanjing, China; Cat. No. C016‐1‐1) was used for this assay. α‐amylase hydrolyzes starch to produce glucose, maltose, and dextrin. The unhydrolyzed starch forms a blue complex with iodine solution, and the intensity of the blue color can be used to calculate the content of α‐amylase. Experimental procedures: The colorimetric tank of the spectrophotometer was preheated at 37°C in advance. The substrate solution (starch) was preheated at 37°C for more than 5 min. The crude enzyme solution mixed with substrate solution was added and incubated at 37°C in a water bath for 7.5 min. Subsequently, iodine solution was added, and the mixed liquid was poured into a cuvette to measure the absorbance value at a wavelength of 660 nm. The absorbance value of the mixture without adding the enzyme solution was used as a blank control to calculate the enzyme activity. The catalytic production of 1 μg of reducing sugar per minute by cellulase in 1 mg of protein was defined as one unit of enzyme activity (U/mg prot).

#### 2.2.5 Assessment of Cellulase Activity

The cellulase assay kit (Nanjing Jiancheng, Nanjing, China; Cat. No. A138) was used for this assay. Cellulase hydrolyzes cellulose to produce reducing sugars, such as cellobiose and glucose, which can reduce 3,5‐dinitrosalicylic acid (DNS) under alkaline conditions to produce a brownish‐red amino compound. The maximum light absorption occurs at a wavelength of 550 nm, and the activity of cellulase can be determined by measuring the amount of reducing sugars produced using colorimetry. Experimental procedures: A portion of the crude enzyme solution was boiled in water for 5 min (to inactivate the enzyme) and used for the control tube. The untreated crude enzyme solution was used for the assay tube. Add the crude enzyme solution, buffer solution, substrate solution (cellulose), and distilled water into the assay tube and incubate it at 37°C for 30 min. Then, immediately immerse the tube in boiling water for 15 min. After cooling, centrifuge the mixture (4000 rpm, at room temperature) and retain the supernatant. Except for the addition of boiled crude enzyme solution, the other steps of the control tube were the same as those of the assay tube. The supernatant was mixed with the DNS test solution and then reacted in a boiling water bath for 15 min. After cooling, add distilled water and pour the mixture into a cuvette to measure the absorbance value at a wavelength of 550 nm. The enzyme activity can be obtained by calculation. The catalytic production of 1 μg of reducing sugar per minute by cellulase in 1 mg of protein was defined as one unit of enzyme activity (U/mg prot).

#### 2.2.6 Statistical Analysis

All data analyses were completed using SPSS 25.0, significant differences between LC group and FG group were tested by a two‐tailed paired *t* test. Values of *p* < 0.05 were considered statistically significant.

### 2.3 DNA Extraction and PCR Amplification

Total bacterial DNA was extracted from the fecal samples using a TGuide S96 Magnetic Soil and Stool DNA Kit (TIANGEN, Beijing, China; Cat. No. DP812). The quantity and quality of the extracted DNA were measured using a microplate reader (Biotek Synergy HTX; Agilent Technologies Inc., Santa Clara, CA, USA) and 1.8% agarose gel electrophoresis, respectively.

The full‐length 16S rRNA genes were amplified with the primer pairs 27F: AGRGTTTGATYNTGGCTCAG and 1492R: TASGGHTACCTTGTTASGACTT. Both the forward and reverse 16S primers were tailed with sample‐specific PacBio barcode sequences to allow multiplexed sequencing. We chose to use barcoded primers because this method reduces chimera formation in comparison with the alternative protocol in which primers are added in a second PCR reaction. The KOD One PCR Master Mix (TOYOBOLife Science) was used to perform 25 cycles of PCR amplification, with initial denaturation at 95°C for 2 min, followed by 25 cycles of denaturation at 98°C for 10 s, annealing at 55°C for 30 s, extension at 72°C for 1 min 30 s, and a final step at 72°C for 2 min. All of the PCR amplicons were purified with VAHTSTM DNA Clean Beads (Vazyme, Nanjing, China) and quantified using the Qubit dsDNA HS Assay Kit and Qubit 3.0 Fluorometer (Invitrogen, Thermo Fisher Scientific, Oregon, USA). After the individual quantification step, the amplicons were pooled in equal amounts. SMRTbell libraries were prepared from the amplified DNA using the SMRTbell Express Template Prep Kit 2.0 according to the manufacturer's instructions (Pacific Biosciences). Purified SMRTbell libraries from the pooled and barcoded samples were sequenced on a PacBio Sequel II platform (Beijing Biomarker Technologies Co. Ltd., Beijing, China) using the Sequel II binding kit 2.0.

### 2.4 Bioinformatic Analysis

Raw subreads were calibrated to obtain circular consensus sequencing (CCS) (SMRT Link, version 8.0), and Lima (v1.7.0) software was used to identify the CCS sequences of different samples through barcode sequences and to remove chimeras to obtain effective CCS sequences. The operational taxonomic units (OTUs) were clustered with a 97% similarity cutoff using UPARSE (version 10.0) (Edgar 2013).

Alpha diversity index analysis was performed using QIIME2 (version 2020.6) software (Bolyen et al. 2019), and the Wilcoxon rank sum test was used to compare community diversity indices (Ace richness estimator and Shannon–Wiener index) (Prehn‐Kristensen et al. 2018).

The dimensionality reduction in the data was based on the Bray–Curtis distance and the binary Jaccard distance matrix using principal component analysis (PCA) and nonmetric multidimensional scaling (NMDS). This was used to observe differences in the gut microbial community structure (Oksanen et al. 2016). In addition, permutational multivariate analysis of variance (PERMANOVA) and analysis of similarities (ANOSIM) (Anderson and Walsh 2013) were conducted using the R v3.1.1 package “vegan” (v2.3–0) (Oksanen et al. 2016) to assess the significance of the differentiation of the microbiota structures among the groups.

Using SILVA 132 (Quast et al. 2013) as the reference database, the community species composition analysis of the two groups of experiments was performed at various levels: phylum, order, family, and genus. Linear discriminant effect size (LEfSe) analysis was used to screen the microorganisms with large differences as potential markers (Segata et al. 2011); the significance of the different species was determined using Matestats software, and differential strains were screened according to the criteria of LDA > 4, *q* < 0.05. PICRUSt2 (Douglas et al. 2019) software was used to compare the 16S sequencing data to obtain species composition information based on the Kyoto Encyclopedia of Genes and Genomes (KEGG) database; the gene functions corresponding to the sequencing data were obtained and corresponded to the KEGG pathways. The significance of the functional pathways in the two groups of samples was compared using the *t*‐test in STAMP and the *p*‐value threshold of 0.05.

### 2.5 LC–MS Metabolomics Detection

A fecal sample (50 mg) was weighed, and 1000 μL of the solution containing an internal standard (1000:2) (methanol acetonitrile: water = 2:2:1, internal standard concentration 2 mg/L) was added; then, the sample was vortexed for 30 s. Subsequently, magnetic beads were added, ground at 45 Hz for 10 min, sonicated in an ice water bath for 10 min, left to stand for 1 h, and centrifuged at 4°C, 12,000 rpm for 15 min. A 500 mL aliquot was placed in a centrifuge tube and dried under vacuum. After drying, 160 μL of the solution (acetonitrile: water = 1:1) was added, vortexed for 30 s, sonicated for 10 min in an ice water bath, and centrifuged at 12,000 rpm for 15 min at 4°C. Finally, 120 μL of the supernatant was collected, and 10 μL was taken from each sample to be mixed into QC samples for machine testing.

Metabolites in the fecal samples were detected in positive and negative ion modes and separated using a Waters Acquity 1‐Class PLUS ultraperformance liquid chromatography system (XevoG2‐XS QTOF high‐resolution mass spectrometer; Waters Corp., Milford, MA, USA). Primary and secondary mass spectrometry data were collected in MSe mode under the control of acquisition software (MassLynx V4.2; Waters). In each data acquisition cycle, dual‐channel data acquisition was performed simultaneously at both low and high collision energies. The low collision energy was 2 V, the high collision energy range was 10–40 V, and the scI ion source was as follows: capillary voltage, 2000 V (positive ion mode) or −1500 V (negative ion mode); cone voltage, 30 V; ion source temperature, 150°C; desolvent gas temperature, 50°C; backflush gas flow rate, 50 L/h; and desolventizing gas flow rate, 800 L/h.

The raw data collected using MassLynx V4.2 were processed using Progenesis QI software for peak extraction, peak alignment, and other data processing operations, based on the Progenesis QI software online METLIN database and Biomark's self‐built library for identification; at the same time, theoretical fragment identification and mass deviation were within 100 ppm.

### 2.6 Quality Control and Data Analysis of Fecal Metabolite Detection

Principal component analysis (PCA) of the metabolite data was performed, and the PLS‐DA and OPLS‐DA model was calculated using the R (3.3.2) package “ropls” to visualize sample clustering (Thevenot 2016). Differential metabolites were screened by combining the fold difference, the *p*‐value of the *t*‐test, and the VIP value of the OPLS‐DA model. The screening criteria were FC > 1, *p*‐value < 0.05, and VIP > 1. The cluster profiler used the hypergeometric test method to perform enrichment analysis of the annotation results of the differential KEGG metabolites and to screen the representative metabolic pathways for their impact value and their significance as enrichment criteria.

The metabolome data were preprocessed by UV scaling using R (v 3.6.1), and the metabolite data were dimensionalized using hierarchical cluster analysis (HCA). The metabolites were divided into different metabolite clusters, and the data with a correlation *p*‐value satisfying CCP < 0.05 were retained. The NbClust package selected the best number of metabolite clusters, the R hclust function performed clustering, and the metabolite clusters were determined using the R cutree function. The Hmisc package performed correlation analysis, and the pheatmap package performed correlation map visualization.

## 3 Result

### 3.1 Analysis of Gut Microbiota Diversity

**FIGURE 2:**
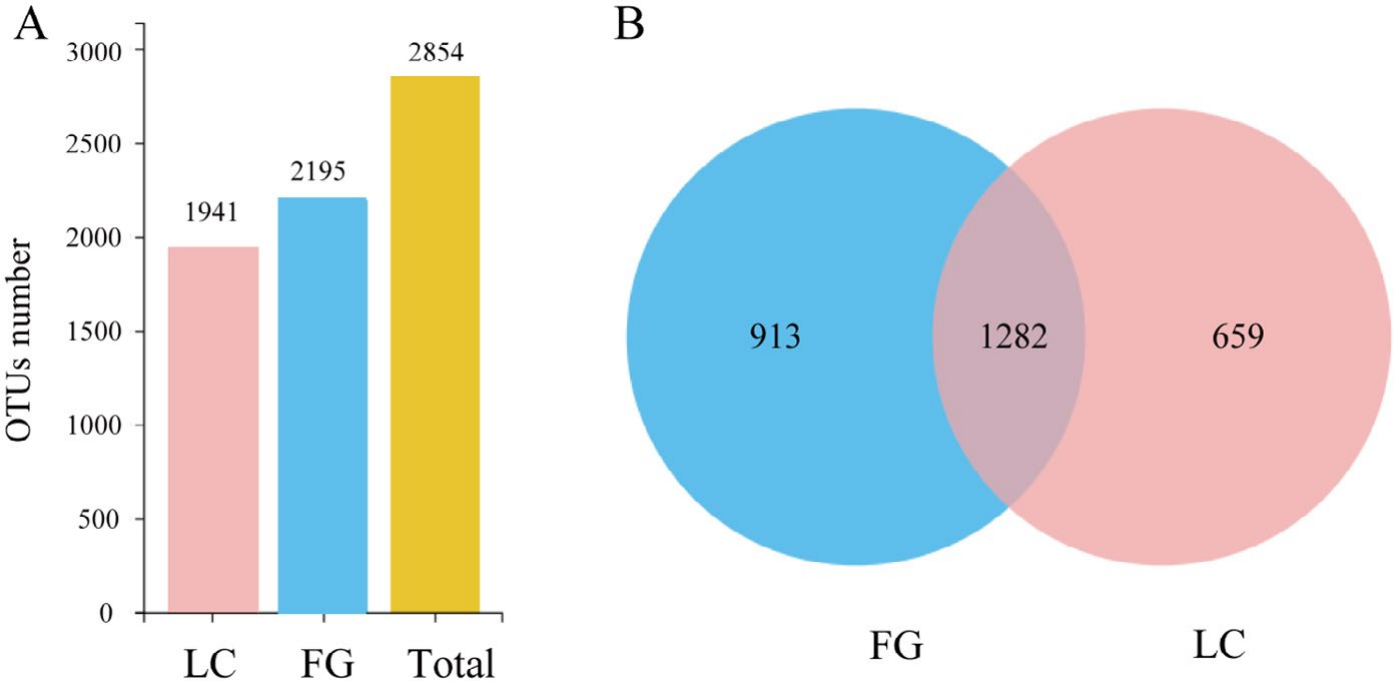
Histogram (A) and Venn plot (B) of gut microbiota OTUs of *T. roborowskii* in LC and FG group.

A total of 2854 different OTUs were classified into the LC and FG groups. The number of OTUs present in both the LC and FG groups was 1282, with 659 unique OTUs in the LC group, and 913 unique OTUs in the FG group (Figure 2).

The alpha diversity indices, including ACE, Chao1, Simpson, and Shannon, are shown in Table 2. There were no significant differences in alpha diversity between the two groups at the OTU, phylum, and genus level. However, at the order level, the Simpson index and Shannon index in the LC group were significantly higher than those in the FG group (*p* < 0.05), and the ACE index in the LC group was also significantly higher than that in FG group at the family level (*p* < 0.05).

**TABLE 2:** Alpha diversity index of intestinal flora at different taxonomic levels.

Group	Ace	*p*	Chao 1	*p*	Simpson	*p*	Shannon	*p*
LC (OTU)	574.05 ± 89.11	0.15	582.03 ± 87.04	0.13	0.97 ± 0.01	0.38	6.6 ± 0.46	0.96
FG(OTU)	702.69 ± 150.90		723.22 ± 157.47		0.96 ± 0.04		6.62 ± 0.79	
LC (phylum)	11.8 ± 1.30	0.67	11.8 ± 1.30	0.42	0.6 ± 0.11	0.69	1.84 ± 0.34	0.29
FG (phylum)	11.47 ± 0.98		11.2 ± 0.84		0.57 ± 0.16		1.58 ± 0.39	
LC (order)	41.06 ± 4.88	0.082	40.9 ± 6.41	0.12	0.86 ± 0.05	**0.008**	3.34 ± 0.31	**0.01**
FG (order)	35.48 ± 3.87		34.97 ± 3.62		0.75 ± 0.05		2.68 ± 0.31	
LC (family)	69.91 ± 4.36	**0.048**	69.97 ± 7.32	0.085	0.9 ± 0.02	0.073	3.98 ± 0.17	0.089
FG (family)	62.04 ± 5.99		62.16 ± 4.66		0.86 ± 0.04		3.6 ± 0.39	
LC (genus)	117.32 ± 9.26	0.68	118.48 ± 10	0.68	0.92 ± 0.02	0.11	4.54 ± 0.17	0.2
FG (genus)	119.84 ± 9.69		120.92 ± 7.7		0.88 ± 0.05		4.15 ± 0.56	

*Note:* The comparison of alpha diversity between LC and FG group, bold numbers showed values with significant differences (*p <* 0.05).

**FIGURE 3:**
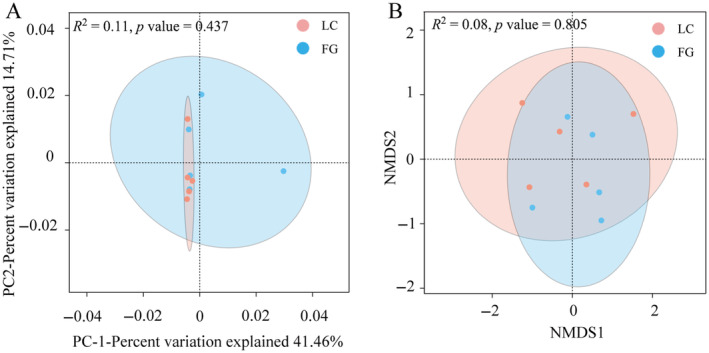
Beta diversity analysis of gut microbiota from *T. roborowskii* according to PCA analysis and NMDS analysis. (A) PCA analysis of the gut microbiota in LC and FG groups based on Bray‐Curtis distance, the discrete distribution of samples along the PC1 and PC2 axes. (B) NMDS analysis of the gut microbiota in LC and FG groups based on Binary‐Jaccard distance.

The PCA and NMDS plots (Figure 3) showed that the LC group overlapped with the FG group and that there was no significant difference between the two groups. However, the distribution within the group in the PCA plots was more discrete in the FG group and more uniform in the LC group, suggesting that the addition of grapes to the diet affected the aggregation and stability of the gut microbiota in captive *T. roborowskii*.

### 3.2 Gut Microbiota Composition in LC and FG *T. roborowskii*

**FIGURE 4:**
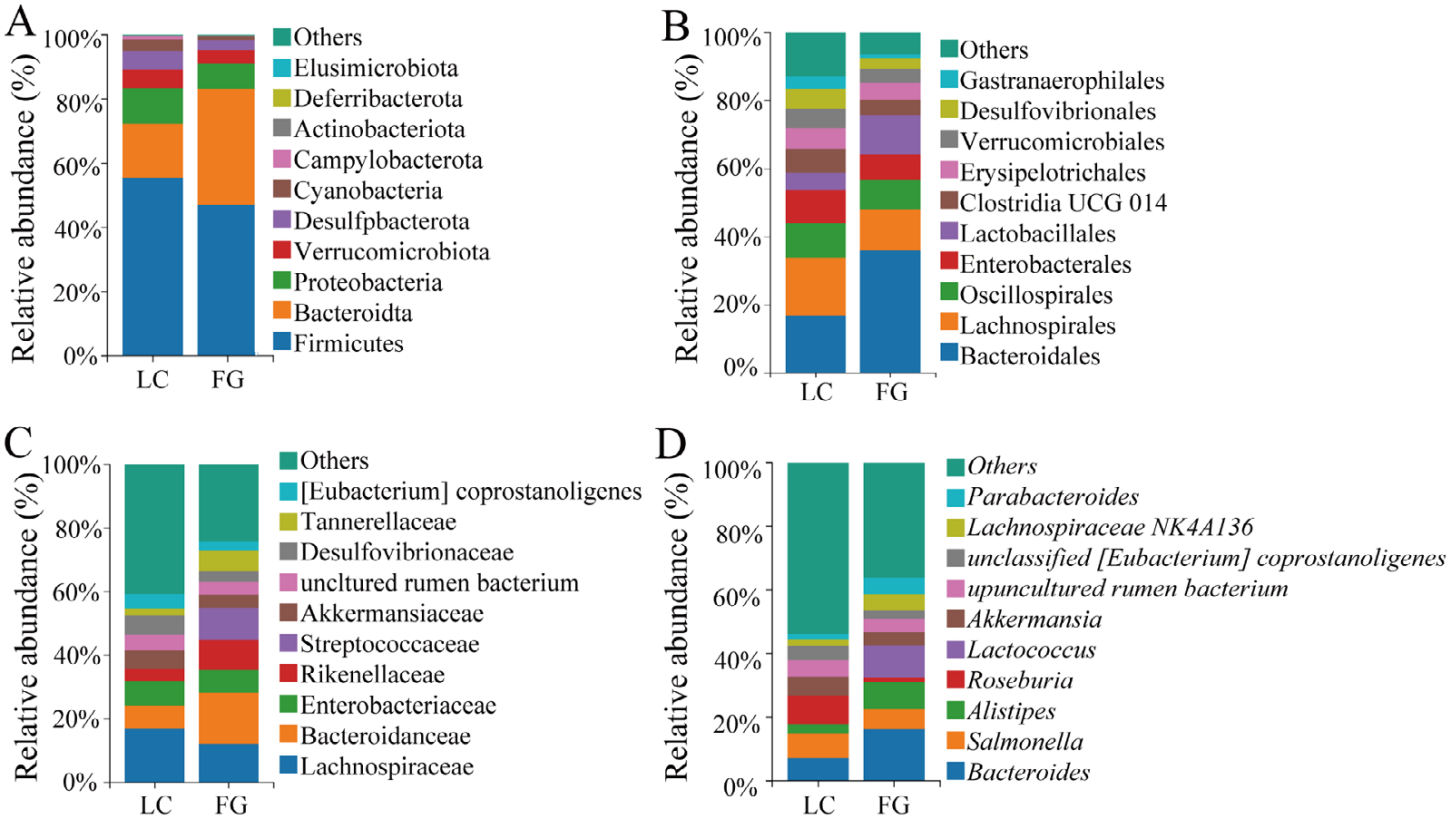
The relative abundance of gut microbiota at the phylum (A), order (B), family (C) and genus (D) levels in LC and FG groups of *T. roborowskii* . The histogram shows the top 10 bacterial phyla, families and genera with relative abundance. The vertical coordinates of the figure indicate the relative abundance of bacterial species and the species names are shown in the legend on the right.

The proportions of microbiota under the different taxonomic classifications are shown in Figure 4. At the phylum level, the gut microbiota was mainly dominated by Firmicutes (55.38%, 47.05%), Bacteroidetes (16.86%, 36.20%), and Proteobacteria (11.09%, 7.76%) in the LC and FG groups, while the other representative phyla included Verrucomicrobiota (5.83%, 4.13%), Desulfobacterota (5.80%, 3.27%), and Cyanobacteria (3.72%, 1.11%) (Figure 4A).

At the order level, in the LC group, the dominant orders were Lachnospirales (17.04%), Bacteroidales (16.84%), and Oscillospirales (10.16%), whereas the other orders with quantitative advantages included Enterobacterales (9.87%), Clostridia_UCG_014 (6.82%), and Erysipelotrichales (6.04%). However, in the FG group, the dominant orders were Bacteroidales (36.19%), Lachnospirales (12.05%), and Lactobacillales (11.65%), followed by a higher abundance of Oscillospirales (8.62%), Enterobacterales (7.26%), and Erysipelotrichales (5.01%) (Figure 4B).

At the family level, in the LC group, Lachnospiraceae (16.95%), Enterobacteriaceae (7.76%), and Bacteroidaceae (7.19%) were predominant; Akkermansiaceae (5.82%) and Desulfovibrionaceae (5.80%) were the other four major families. As for the FG group, the most abundant taxa were Bacteroidaceae (16.23%), Lachnospiraceae (12.03%), and Streptococcaceae (10.21%), followed by a higher abundance of Rikenellaceae (9.46%), Enterobacteriaceae (7.15%), and Tannerellaceae (6.45%) (Figure 4C).

At the genus level, the gut microbiota of *T. roborowskii* in the LC group was dominated by *Roseburia* (9.13%), *Salmonella* (7.74%), and *Bacteroides* (7.19%); the other predominant genus was *Akkermansia* (5.82%). Compared with the LC group, the dominant genus in the FG group included *Bacteroides* (16.23%), *Lactococcus* (10.21%), and *Alistipes* (8.33%), while the other identifiable genera with a relative abundance of > 5% included *Salmonella* (6.49%), *Parabacteroides* (5.21%), and *Lachnospiraceae NK4A136* (5.05%) (Figure 4D).

### 3.3 Predictive Analysis of Gut Microbial Function

**FIGURE 5:**
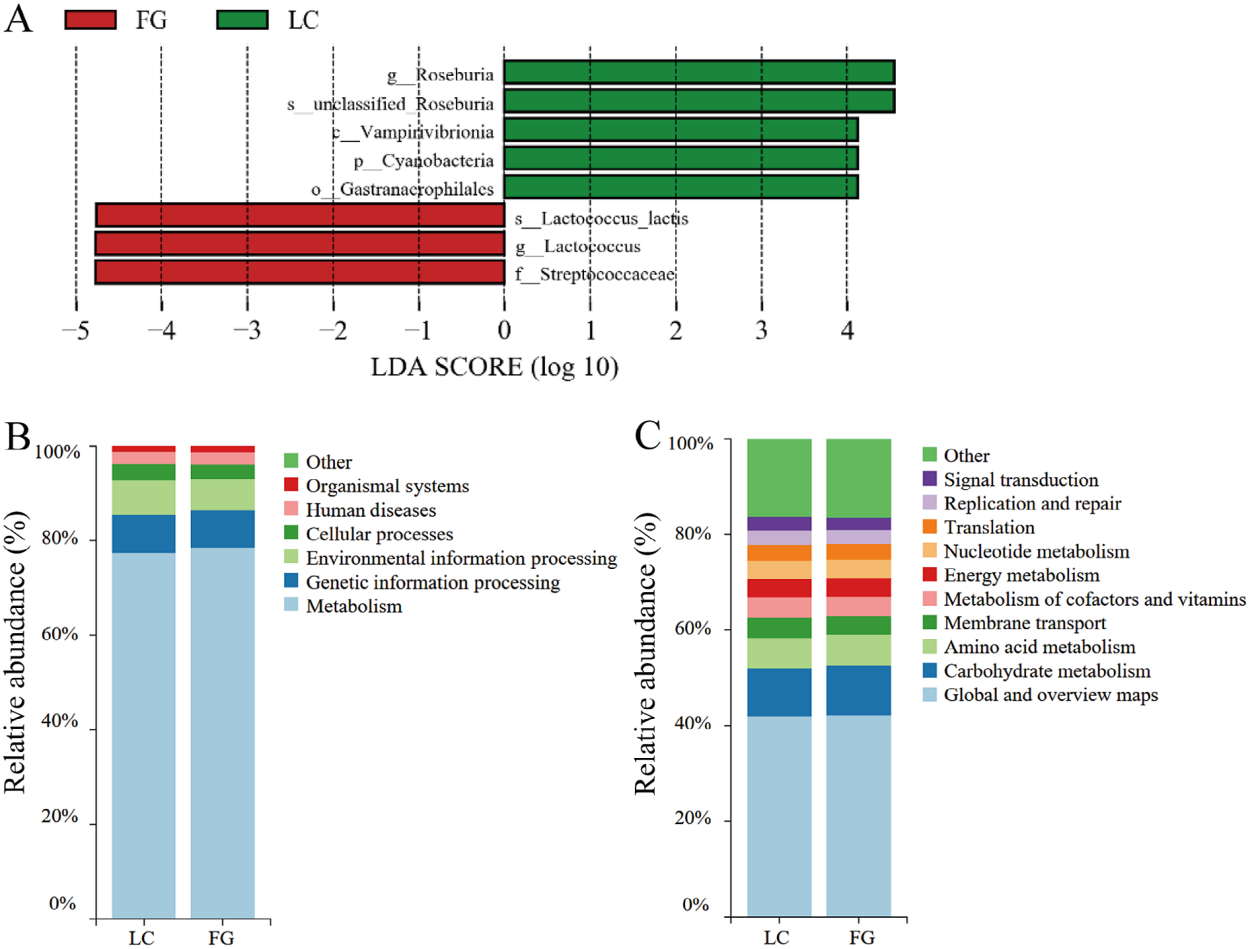
LEfSe and KEGG pathways analysis of gut microbiota in LC and FG groups. (A) LEfSe analysis of gut microbiota composition in LC and FG groups of *T. roborowskii* (LDA > 4, *p* < 0.05). The highlighted taxa were significantly enriched in the group that corresponds to each color. The letters “p”, “c”, “o”, “f”, “g,” and “s” indicate phylum, class, order, family, genus, and species, respectively. Histogram of KEGG functional composition of gut microbes in *T. roborowskii* in LC and FG group at the top level (B) and second level (C).

In addition, LEfSe analyses were performed between the two groups to estimate the difference in relative abundance at the different bacterial taxonomic levels (LDA > 4, *p* < 0.05) (Figure 5A). The relative abundances of Cyanobacteria at the phylum level, Vampirivibrionia at the class level, Gastranaerophilales at the order level, and *Roseburia* at the genus level in the LC group were significantly higher than in the FG group. However, the relative abundance of Streptococcaceae at the family level, *Lactococcus* at the genus level, and *Lactococcus lactis* at the species level in the FG group was significantly higher than that in the LC group.

The functional pathway abundance of KEGG, corresponding to the 16S rRNA sequencing data, was predicted using PICRUSt2. Notably, the majority of the resultant KEGG categories belonged to metabolism, genetic information processing, environmental information processing, cellular processes, human diseases, and organismal systems at the first level (Figure 5B); the global and overview maps, carbohydrate metabolism, and amino acid metabolism were enriched in the second‐level metabolic pathways, but there were no significant differences between the two groups (Figure 5C).

### 3.4 Differential Analysis and Functional Annotation of Fecal Metabolites in LC and FG *T. roborowskii*

**FIGURE 6:**
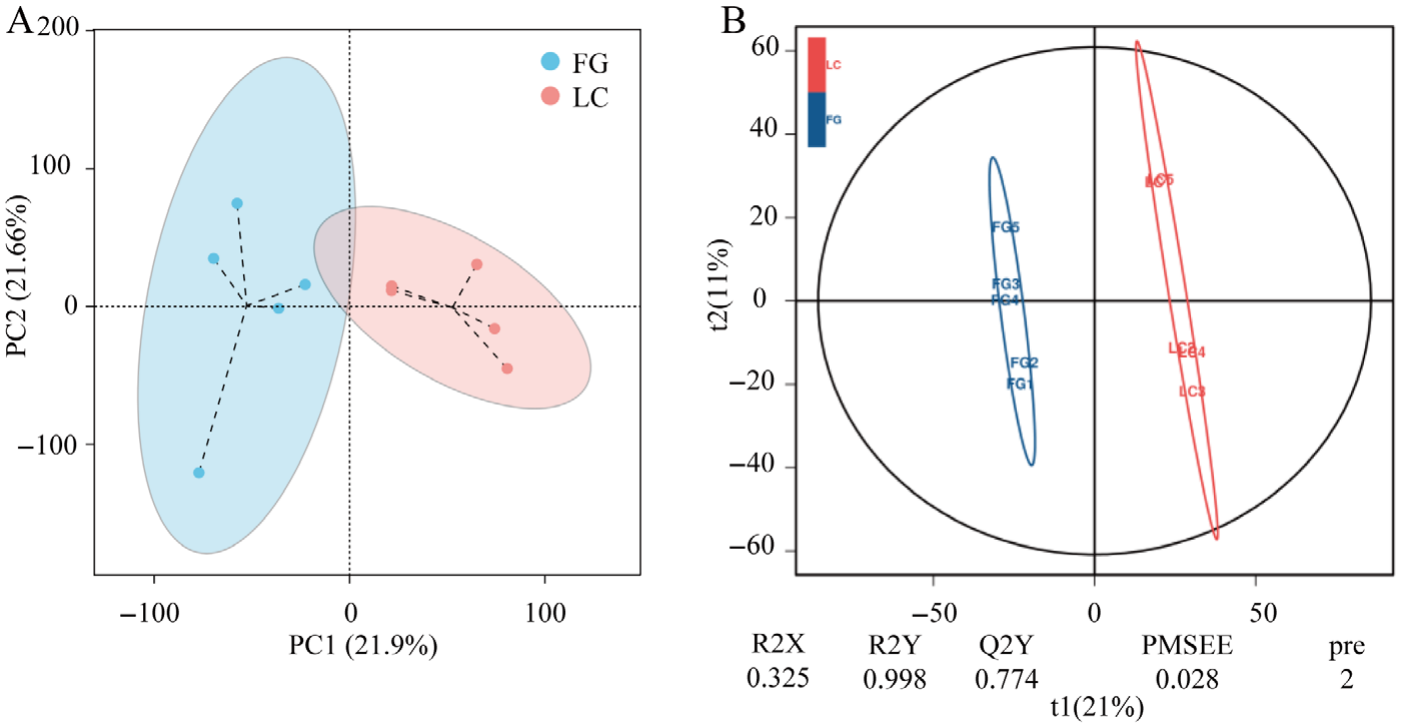
PCA and PLS‐DA score plots in LC and FG groups. (A) PCA analysis on fecal metabolites of LC and FG *T. roborowskii*. (B) PLS‐DA analysis of fecal metabolites.

Based on the PCA analysis, a superior separation was observed between the two groups, which revealed a remarkable alteration of metabolites in the *T. roborowskii* (Figure 6A). The PLS‐DA model revealed that the fecal metabolites of the two groups could be significantly distinguished, indicating that the metabolic profiles of the LC group were different from those of the FG group (Figure 6B).

**FIGURE 7:**
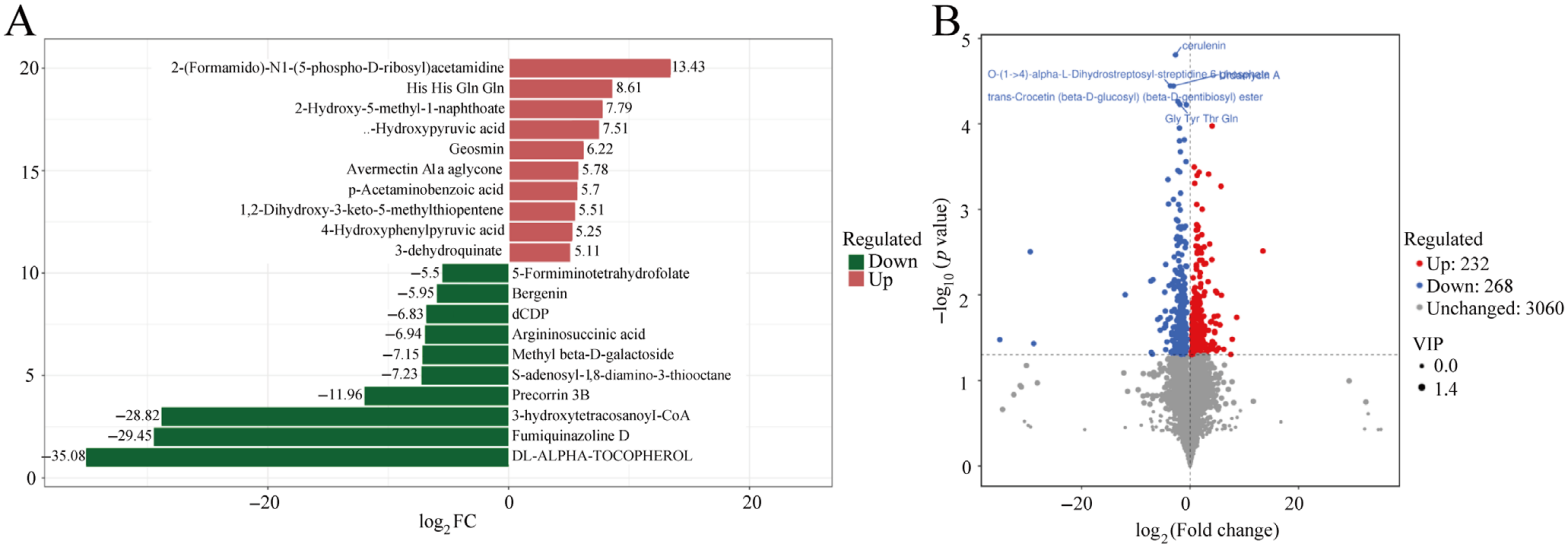
Difference analysis of fecal metabolites of *T. roborowskii*. (A) Fold Change analysis of fecal metabolites differences based on log_2_FC size ordering. The figure shows the top 10 metabolites of up and down logFC, and the labels of each column indicate the name of the metabolite. Red bars indicate higher levels in the FG group and green bars indicate higher levels in LC group. (B) Volcano plot for statistical analysis of fecal differential metabolites. Each point in the volcano plot represents a metabolite, the abscissa represents the multiple changes of the group compared with each substance, the ordinate represents the *p*‐value of the *t*‐test, and the size of the scatter represents the VIP value of the OPLS‐DA model.

The absolute values of log_2_FC (Fold Change, FC) were sorted to obtain the top 10 metabolites in each group (Figure 7A). In the LC group, the contents of metabolites such as dl‐alpha‐tocopherol, fumiquinazoline D, 3‐hydroxytetracosanoyi‐coa, precorrin 3B, s‐adenosyl‐1,8‐diamino‐3‐thiooctane, methyl beta‐d‐galactoside, argininosuccinic acid, dCDP, Bergenin, and 5‐formaminotetrahydrofolate were relatively higher. 2‐(Formamido)‐N1‐(5‐phospho‐D‐ribosyl)acetamidine, His His Gln Gln, 2‐Hydroxy‐5‐methyl‐1‐naphthoate, Hydroxypyruvic acid, Geosmin, Avermectin A1a aglycone, p‐Acetaminobenzoic acid, 1,2‐Dihydroxy‐3‐keto‐5‐methylthiopentene, 4‐Hydroxyphenylpyruvic acid, and 3‐dehydroquinate were more abundant in the FG group.

According to the criteria of *p* < 0.05, VIP > 1, and FC > 1, 500 differential metabolites were identified. In the FG group, 232 metabolites were upregulated, and 268 metabolites were downregulated. Specifically, compared with the LC group, the levels of 2‐Phenylacetamide, ethisterone, zymosterol, etc., were significantly increased, while the levels of cerulenin, O‐(1‐> 4)‐alpha‐L‐Dihydrostreptosyl‐streptidine 6‐phosphate, and Urdamycin A were increased in the captive group (Figure 7B).

**FIGURE 8:**
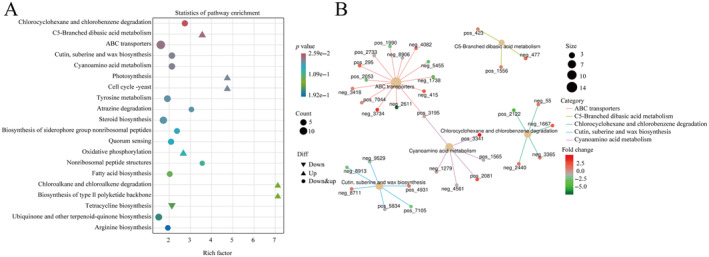
Difference analysis of metabolic functional pathways between LC group and FG group in *T. roborowskii*. (A) Bubble diagram of functional pathways corresponding to differential metabolites of *T. roborowskii* in LC and FG groups. (B) Enrichment of functional pathways corresponding to differential metabolites of *T. roborowskii* in LC and FG groups.

Further analysis of the metabolic pathways of the LC and FG groups of *T. roborowskii* using a bubble diagram revealed the pathways of tryptophan, chlorocyclohexane and chlorobenzene degradation; C5‐Branched dibasic acid metabolism, ABC transporters, etc., were enriched significantly in both groups (Figure 8A). The KEGG enrichment network diagram shows that these differential functional pathways are closely related to the differential metabolites between the two groups, either positively or negatively. Among them, L‐phenylalanine (pos_3195 in Figure 8B) was associated with two functional pathways, suggesting that it may be a key factor affecting metabolic pathways (Figure 8B).

### 3.5 Microbiota‐Metabolome Association

**FIGURE 9:**
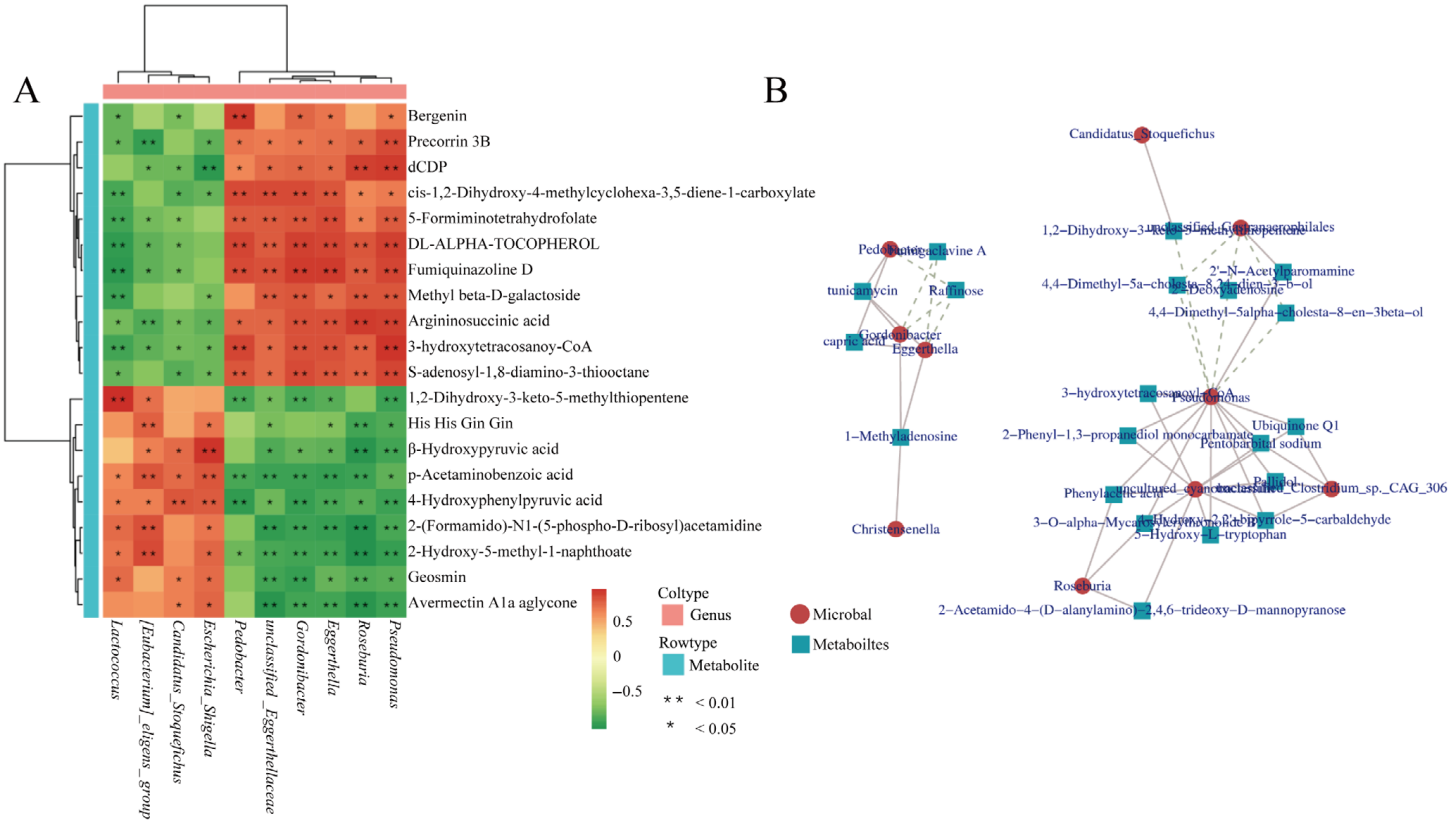
Gut microbiota and metabolites correlation analysis. (A) Correlation analysis between differential genera and top 20 differential metabolites. *p < 0.05. **p < 0.01. (B) Correlation network of different gut microbiota and different metabolites.

**FIGURE 10:**
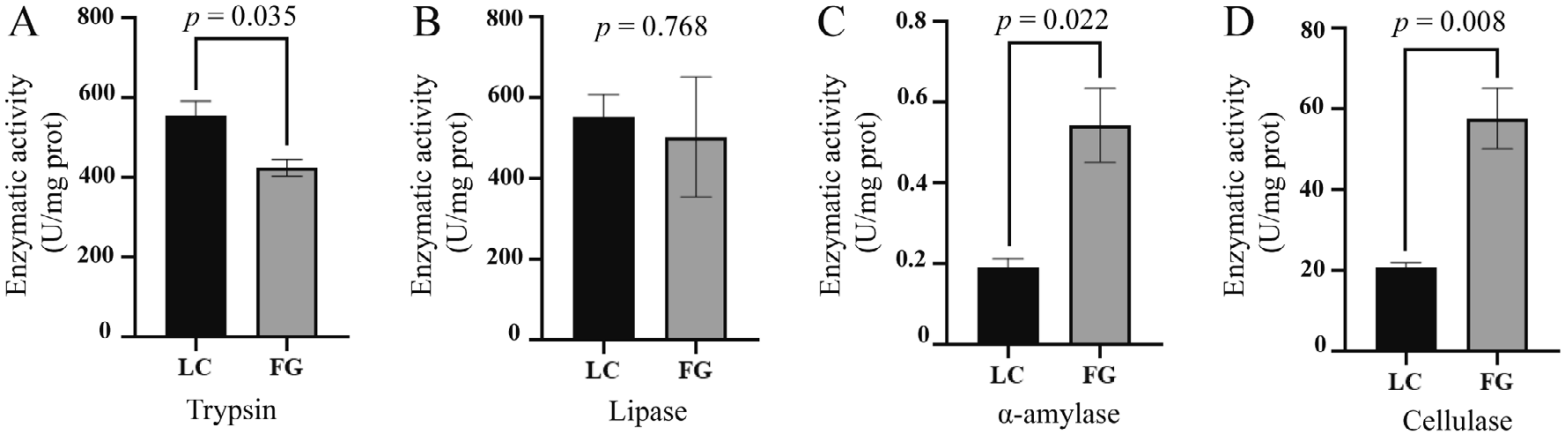
The digestive enzyme activities of *T. roborowskii* between LC group and FG group. Significant differences between LC group and FG group were tested by a two‐tailed paired *t* test, values of *p* < 0.05 were considered statistically significant.

Correlation heatmaps were drawn to explore the relationship between differential metabolites (top 20) and differential microbiota (10 genera) (Figure 10A). The differential microbiota was closely related to the differential metabolites, showing different degrees of positive or negative correlation. *Escherichia Shigella*, *Candidatus Stoquefichus*, *[Eubacterium] eligens*, and *Lactococcus* had a significant positive correlation with p‐acetaminobenzoic acid, 4‐hydroxyphenylpyruvic acid, etc., and a significant negative correlation with argininosuccinic acid, 3‐hydroxytetracosanoyI‐CoA, etc. *Pseudomonas*, *Roseburia*, *Eggerthella*, *Gordonibacter*, and *Pedobacter* had a significant positive correlation with precorrin 3B, dCDP, 5‐formiminotetrahydrofolate, fumiquinazoline D, etc., and a significant negative correlation with p‐acetaminobenzoic acid, 4‐hydroxyphenylpyruvic acid, 2‐hydroxy‐5‐methyl‐1‐naphthoate, etc. (Figure 9A).

The screened differential metabolites were correlated with the differential microorganisms. The correlation network diagram shows that *Pseudomonas* seemed to be the core genus given that it was negatively correlated with a variety of differential metabolites. In addition, *Roseburia*, *Eggerthella*, *Gordonibacter*, *Pedobacter*, *Christensenella*, etc., were closely related to the differential metabolites (Figure 9B).

### 3.6 Analysis of Digestive Enzyme Activity

The digestive enzyme activities in the different groups of *T. roborowskii* showed different characteristics. The α‐amylase and cellulase activities in the FG group were significantly higher than those in the LC group (Figure 10C,D). The trypsin activity in the LC group was remarkably higher than that in the FG group (Figure 10A), while there was no significant difference in lipase activity between the two groups (Figure 10B).

### 3.7 Correlation Analysis of Gut Microbiota, Metabolites, and Digestive Enzyme

**FIGURE 11:**
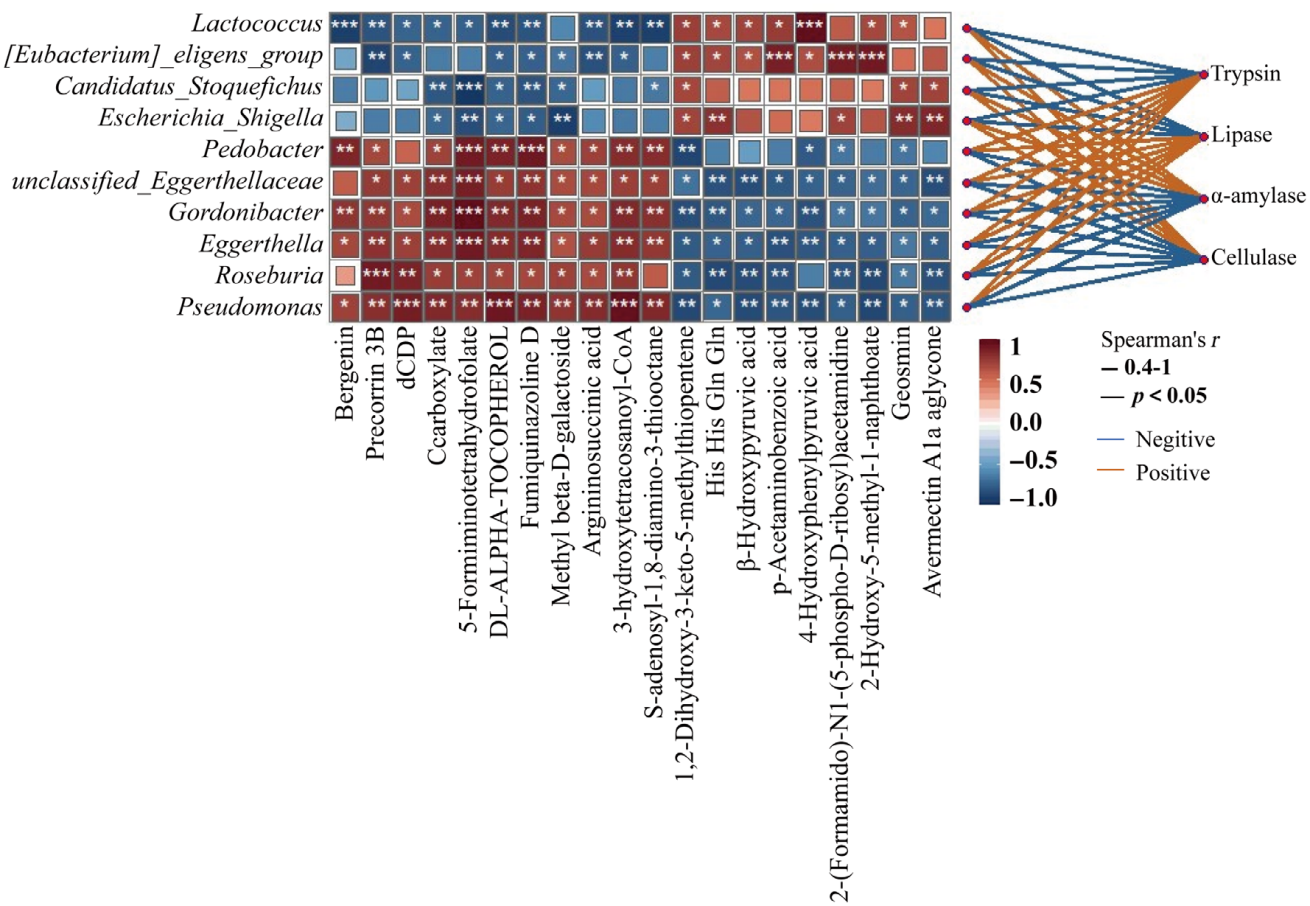
Correlation analysis of differential gut microbiota, differential metabolites, and digestive enzyme activities in *T. roborowskii*. The horizontal axis of the heat map is the name of the digestive enzyme, and the vertical axis is the name of the microorganism, digestive enzyme names are on the far right. The darker the square color, the higher the correlation. Red lines indicate a positive correlation between digestive enzyme activity and microorganisms, while blue lines indicate a negative correlation. “*” indicates significant correlation (*p < 0.05, **p < 0.01, ***p < 0.001).

The correlation of digestive enzyme activities, differential gut microbiota, and differential metabolites between the two groups was analyzed. The differential microbiota was closely related to the differential metabolites, and digestive enzyme activities had different positive or negative correlations with the abundance of different microbiota. Notably, the abundance of *Escherichia Shigella*, *Candidatus Stoquefichus*, *[Eubacterium] eligens*, and *Lactococcus* was significantly positively correlated with α‐amylase and cellulase activities; and *Pseudomonas*, *unclassified Eggerthellaceae*, *Roseburia*, *Eggerthella*, *Gordonibacter*, and *Pedobacter* with trypsin and lipase activities (Figure 11).

## 4 Discussion

The gut microbiota is a complex network of bacteria, fungi, protozoa, archaea, and virus communities, which play an important role in the health of the host (Bestion et al. 2017). The diversity and flexibility of the composition and function of the gut microbiota are key factors in coping with environmental changes (Hauffe and Barelli 2019). Diet is considered to be one of the important driving factors of changes in the composition of animal gut microbiota. For example, omnivorous lizards *Liolaemus ruibali* , which consume an average of only 16% of the plants in the wild, after feeding on a plant‐rich diet, lizards had a more diverse gut community, with significantly higher abundance of Melainabacteria and *Oscillospira* (Kohl et al. 2016). Different geographic populations of *Podarcis siculus* have different feeding habits, with omnivorous populations having a richer diversity of gut microbiota than insectivorous populations (Lemieux‐Labonté et al. 2022). Similarly, *Calotes versicolor* , feeding on a plant‐based diet in the field state, showed a loss of Firmicutes and Proteobacteria after 90 days of semi‐natural feeding on an artificial diet with more abundant protein and fat (Zhang et al. 2022). *T. roborowskii* in traditional captivity were simply given free access to mealworms, vitamin water, and calcium powder. While the addition of grapes in diets, which is novel to our experiment, increased their intake of plant fiber, water, and other nutrients. Our results showed that adding grapes significant increased the abundance of *L. lactis* in *T. roborowskii* , and metabolomics analysis revealed enrichment of glucose metabolism‐related pathways, which confirming that changes in diet can lead to rapid changes in the gut microbiota.

Generally, animals with more diverse diets may have a more diverse gut microbiota (Laparra and Sanz 2010). A more diverse diet can increase the alpha diversity of the gut microbiota by providing a more diverse range of nutrients (Li et al. 2016). In our study, the alpha diversity showed no significant differences at the OTU, phylum, and genus levels between the two groups, while the diversity of the LC group was significantly higher than the FG group at the order level (Simpson and Shannon indices) and family level (Ace index). Some microorganisms have a wide range of nutrients that meet their growth requirements in the intestinal environment, and they gain a competitive advantage to become dominant microorganisms in the gut. However, a small number of microorganisms have specific nutrient profiles that cannot persist in the host gut, which may be one of the reasons why the alpha diversity of the gut microbiota does not increase with dietary diversity (Flint et al. 2015). In addition, since each type of food may contain chemical components that affect the presence, growth, or inhibition of certain microbiota, the diversity of the gut microbiota may not increase with dietary diversity (Li et al. 2016). In natural environments, individuals with more similar diets have more similar gut microbial compositions (Graf et al. 2015). The results of this study indicate that short‐term grape feeding did not significantly alter the beta diversity of *T. roborowskii's* gut microbiota, but the principal coordinate analysis showed that the FG group was more dispersed than the LC group, indicating that the composition of the gut microbiota structure can be regulated by adjusting the diet composition, and this result may also be due to the contingency caused by the low sample size.

The dominant bacterial phyla in the gut microbiota of *T. roborowskii* are similar to those of most reptiles, including *Sceloporus grammicus* (Montoya‐Ciriaco et al. 2020), *Phrynocephalus vlangalii* (Zhang et al. 2018), and *Iguana iguana* (Hong et al. 2011), and mainly consist of Firmicutes and Bacteroidetes. Firmicutes and Bacteroidetes cooperate with bacteria that specialize in fermenting oligosaccharides, such as *Bifidobacterium*, to produce short‐chain fatty acids from indigestible carbohydrates (Marchesi et al. 2016). The Proteobacteria phylum is a common and potentially pathogenic group of bacteria in the gastrointestinal tract that have a direct impact on host health through changes in abundance (Shin, Whon, and Bae 2015). These include *Salmonell*a in Proteobacteria, which has certain pathogenic properties and may produce lipopolysaccharides (LPS) and irritating flagellin to promote inflammatory responses (Guo et al. 2022). Naturally, wild animals can be asymptomatic carriers of Salmonella spp., with the bacterium remaining in equilibrium with the intestinal microbiota. When these animals are kept away from their natural habitat, the resulting stress compromises their immune system and destabilizes the microbiota, leading to increased elimination of the pathogen in feces. Therefore, wild animals kept in captivity tend to have a higher prevalence of *Salmonella* (dos Santos, Lopes, and Maciel 2022). In captivity, constant cohabitation, social interaction, and interaction with human keepers provide increased opportunities for transmission of microbiota from host‐associated sources, which are capable of colonizing the animals. The captive population of lizard has more opportunities in contacting with humans, by frequent feeding, cleaning of the feeding box, examination of diseases, etc., which may result in colonization of the *Salmonella* from human. Our results showed that *Salmonella* was the dominant bacterium in the LC group, possibly due to exposure to human activities or captivity, which can affect host gut health, leading to gut dysfunction or disease (Zhou et al. 2020). The dominant genera in the FG group were *Bacteroides*, *Lactococcus*, and *Alistipes*. *Lactococcus* is known as a typical probiotic, and *Alistipes* was reported as a producer of acetate and propionate salts in SCFAs, which could play a beneficial role in gut health (Rau et al. 2018). Therefore, the diets of *T. roborowskii* become more varied through the addition of grapes, which may promote gut health. Furthermore, the abundance of *Bacteroides* in the FG group was obviously increased in comparison with the LC group. The primary biological function of *Bacteroides* is the degradation of biopolymers, especially polysaccharides, in the large intestine. The recent sequencing of Bacteroidetes genomes confirms the presence of numerous carbohydrate‐active enzymes covering a large spectrum of substrates of plant, algal, and animal origin (Thomas et al. 2011). Therefore, the higher carbohydrate intake caused by the addition of grapes to the diet may be the reason for the increase in the abundance of *Bacteroides* in the FG group.

*Lactococcus lactis* , one of the main members of lactic acid bacteria (LAB), is commonly used in food fermentation, drug production, and feed supplementation and is considered safe (GRAS) by the US Food and Drug Administration (Mao, Wu, and Wang 2016). This probiotic has been shown to have a positive impact on human and animal gut health. The health‐promoting effects of LAB strains include anti‐inflammatory properties, stimulation of the host immune system, competition with pathogenic bacteria for nutrients and niches, inhibition of the activity of toxic substances, and reduced lactose intolerance (Saxelin et al. 2005; Markowiak and Slizewska 2017). LEfSe analysis showed that the relative abundance of *L. lactis* significantly increased after short‐term feeding with grapes, suggesting beneficial aspects of grapes as food ingredients in *T. roborowskii* . Many studies have reported that grapes exhibit a variety of biological activities, such as antioxidant, gut‐microbiota regulating, cardioprotective, antidiabetic, and anticancer activities (Zhou et al. 2022). The health benefits of grapes are largely attributed to their rich bioactive compounds, especially polyphenols. Among the beneficial effects of polyphenols in body health, special attention is currently paid to their capacity to modulate the composition and metabolic activity of gut microbiota, which is associated with an increase in probiotics and prebiotics (Zorraquín et al. 2020). In general, the intake of grape phenolic compounds seems to increase the abundance of beneficial bacterial species in the guts of animals, including *Bifidobacterium*, *Lactobacillus*, and others (Zorraquín et al. 2020). Studies on mammals (lambs and pigs) have shown that grape extracts can inhibit or reduce pathogenic bacterial communities and increase the abundance of probiotic communities (Kafantaris et al. 2017; Fiesel et al. 2014). The mechanisms of these beneficial effects may be related to the stimulation of probiotic growth by grape phenolic metabolites and their antagonistic effects against pathogens. Therefore, we speculate that the widespread emergence of probiotics may be related to the intake of grapes.

Microbial communities can change their functions rapidly and appropriately in response to dietary changes (David et al. 2014). Here, we found that the metabolism of the gut microbiota in *T. roborowskii* was also changed after the addition of grapes to their diet. The metabolites related to glucose metabolism, such as hydroxyacetone acid and 4‐hydroxyphenylacetone acid, were significantly enriched in the FG group. Excessive amounts of carbohydrates are converted into pyruvate through glucose metabolism, resulting in the production of hydroxyacetone acid. As a result, the metabolites related to glucose metabolism in the FG group were enriched significantly due to the intake of a large amount of sugar in the grapes. Moreover, the differential metabolite analysis showed that zymosterol was significantly enriched in the FG group. Zymosterol is an intermediate metabolite in the cholesterol synthesis pathway (Rosenfeld et al. 1954). It has been reported that proanthocyanidins extracted from grape seed can alter steroid secretion and that they exhibit a cholesterol‐lowering effect (Quifer‐Rada et al. 2016). Thus, the increase in zymosterol content in the FG group may be due to the anthocyanins in grapes that interfere with cholesterol synthesis, leading to a reduction in endogenous cholesterol synthesis and the accumulation of its intermediate product, zymosterol. The ABC transporter function pathway is closely related to a variety of differential metabolites, which could be inhibited by plant secondary metabolites, such as alkaloids, phenolic compounds, and terpenoids. Polyphenols interact with proteins through hydrogen bonds and ionic bonds in amino acid side chains, which affect the three‐dimensional structure of the protein and inhibit its activity (Wink, Ashour, and El‐Readi 2012). Therefore, the addition of grapes to the diet may be an important factor in the significant differences in the ABC transporter pathways between the two groups. The C5‐branched dibasic acid metabolism pathway is involved in carbohydrate metabolism and energy supply and is also associated with SCFAs synthesis (Kingkaw et al. 2023). SCFAs are the products of colonic bacterial degradation of unabsorbed starch and non‐starch polysaccharide (fiber), that is highly enriched in the colonic environment (Bergman 1990). The potential beneficial effects of SCFAs are manifested in the regulation of immune function (Belkaid and Hand 2014), providing energy source (Bergman 1990), and regulating intestinal morphology and function (Scheppach 1994), etc. Therefore, enrichment of the C5‐branched dibasic acid metabolism pathway may promote gut health. In a comparison of the microbial metabolic functions in the feces of healthy and diarrheic calves, the C5‐branched dibasic acid metabolism pathway was significantly enriched in healthy calves (Gomez et al. 2017). The addition of probiotics to the diet increased the abundance of LAB in the intestines of weaned pigs, and the C5‐branched dibasic acid metabolism pathway was obviously enriched (Lu et al. 2019). In our study, we found that the C5‐branched dibasic acid metabolism pathway was significantly upregulated in the FG group, indicating that grapes play an active role in the diet of captive *T. roborowskii* .

Integration of metagenomics and metabolomics could unravel a potential link between microbial metabolites and diverse disease states (Feng et al. 2016). Such an inclusive analysis may reflect a proper assessment of gut metabolites which consequently would expand the potential of metabolomics to understand the metabolic capacity and adaptability of the gut microbes (Lamichhane et al. 2018). According to the correlation heatmap, *Escherichia Shigella*, *Candidatus Stoquefichus*, *[Eubacterium] eligens*, and *Lactococcus* had a common positive or negative correlation with a variety of metabolites, which indicated a certain synergistic ability when performing metabolic functions. The same synergistic relationship also existed in *Pseudomonas*, *Roseburia*, *Eggerthella*, *Gordonibacter*, and *Pedobacter*, while it was completely different from the related metabolites such as *Lactococcus*, suggesting that there is a certain antagonistic relationship between them. Furthermore, the correlation analysis between the metabolites and the gut microbiota revealed that certain specific bacterial groups respond rapidly to the changes in food composition. In particular, *Pseudomonas* was associated with a variety of differential metabolites, including ubiqounone Q1, phenylacetic acid, and 3‐hydroxytetracosanoyl‐CoA. *Pseudomonas* is an opportunistic pathogen (Matar 2018) which is usually found in patients with inflammatory bowel disease, and it contributes to the imbalance of other bacterial communities (Wagner et al. 2008). Compared to the LC group, the *Pseudomonas* abundance decreased in the FG group, indicating that the addition of grapes to the diet might have a beneficial effect on the intestinal health of *T. roborowskii* .

Animals can better adapt to the effects of different nutrients and food quantities in the environment by adjusting their digestive systems appropriately (Karasov and Douglas 2013). Based on dietary regulation studies conducted on different animals, most scholars currently support the “adaptive modulation hypothesis,” which suggests that the concentration of different substrates in food can specifically regulate the activity of the corresponding digestive enzymes in the intestine (Karasov and Diamond 1983). *Podarcis siculus* has two different geographical populations, and the omnivorous population that consumes more plant foods displayed significantly higher amylase activities than the completely insectivorous population (Wehrle et al. 2020). Similarly, a previous study on the intestinal digestive enzymes of fish with different feeding habits showed that digestive enzyme activity was closely related to fish feeding habits. Generally, carnivorous fish have the highest protease and lipase activities, followed by omnivorous fish, and herbivorous fish have the lowest protease and lipase activities, while the amylase activity is the opposite of the protease activity (Du 2019). Our results showed that the activities of α‐amylase and cellulase in the FG group with the mixture of mealworm and grape diets were significantly higher than those in the LC group, and the trypsin activity was shown to be higher in the LC group; these results corresponded with the results of the nutritional composition, further confirming that there should be a match between diet and digestive physiological function of animals. A large proportion of digestive enzymes in the intestine come from the gut microbiota. Studies have shown that the degradation of polysaccharides is one of the main functions of Bacteroidetes, which can produce abundant polysaccharide‐degrading enzymes, such as amylase and cellulase (Brown et al. 2023; Karlsson et al. 2011). Thus, the high abundance of Bacteroidetes might be the major contributor to the higher α‐amylase and cellulase activity in the FG group.

The correlation analysis between the digestive enzymes and the gut microbiota indicated that *L. lactis* , which had a significantly increased relative abundance in the FG group, showed a close positive correlation with α‐amylase and cellulase. Moreover, there was an obvious positive correlation between the increased *Roseburia* (LC group) and trypsin, as well as lipase. These results demonstrated that diet, gut microbiota, and digestive enzymes are closely linked and interact with each other. Although there is no direct evidence to prove whether the changes in digestive enzyme activity are directly caused by these bacteria, they do appear to be a result of host adaptation to dietary changes. The species composition of the gut microbiota has been shown to respond to dietary change. Meanwhile, the metabolic outputs of the microbiota were influenced by the supply of dietary components and via diet‐mediated changes in the microbiota composition. The breakdown of substrates by digestive enzymes in the gut also affects the production of metabolites. Therefore, the adaptive response of the intestinal function caused by diet should be the result of the interaction between the intestinal microbiota, metabolites, and digestive enzymes.

## 5 Conclusion

**FIGURE 12:**
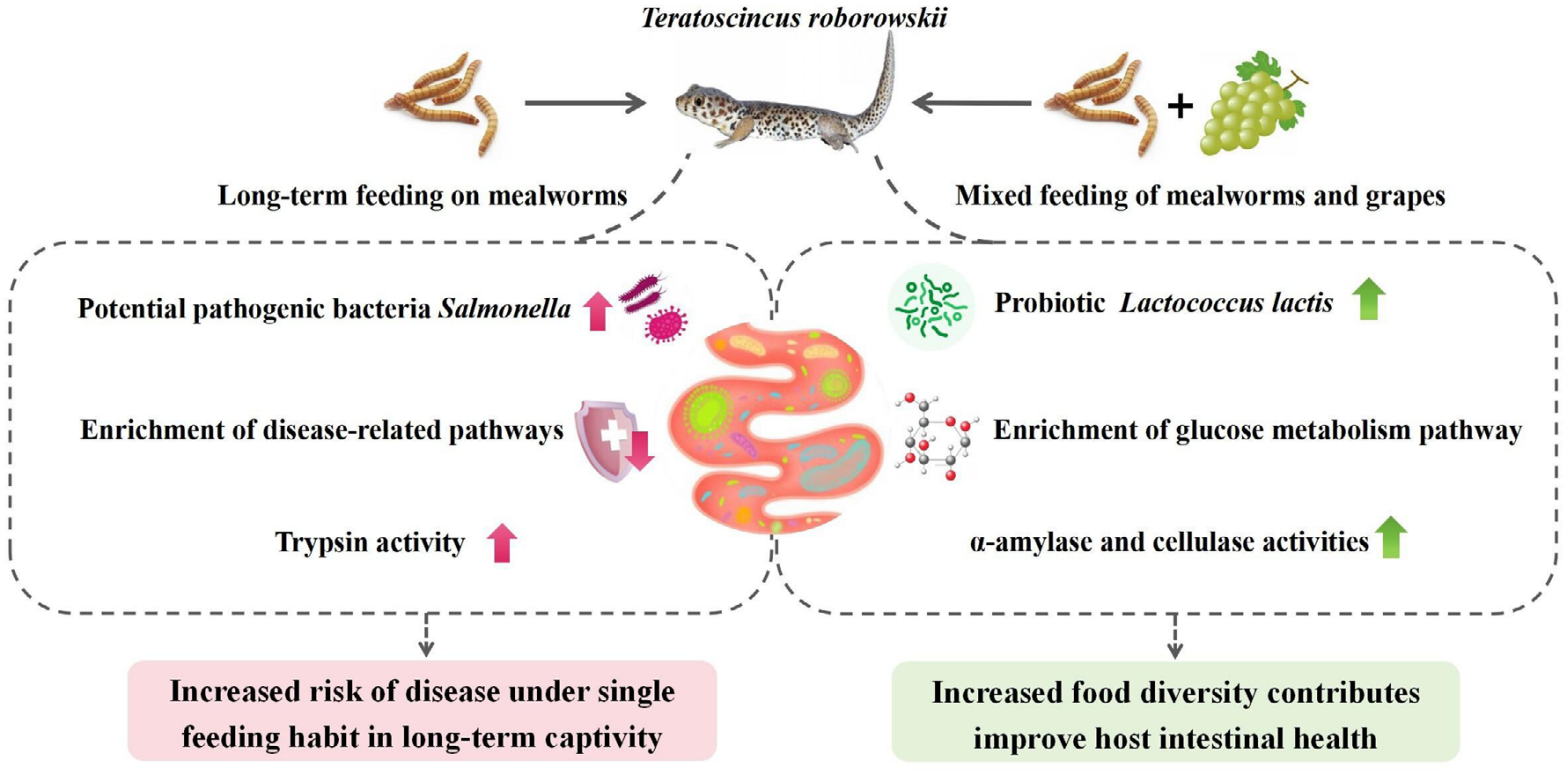
Effects of different diets on *T. roborowskii* .

In summary, the addition of grapes to the diet caused significant changes in the gut microbiota of *T. roborowskii* . Our results indicated that after adding grapes to the diet, there was a notable shift in the microbiota composition, in particular an increase in the beneficial bacteria *Lactococcus* in the FG group. As a result, the pathways related to glucose metabolism in the FG group were significantly enriched. At the same time, the intake of grapes also increased the activity of the amylase and cellulase in *T. roborowskii* , which confirmed that there should be a match between the animal's intestinal function (digestive enzyme activity) and the food it consumes (Figure 12). Our study provides a theoretical basis for the host health and scientific captive breeding of the desert lizards *T. roborowskii* .

## Author Contributions

**Ziyi Wang:** conceptualization (equal), data curation (equal), methodology (equal), resources (equal), writing – original draft (equal), writing – review and editing (equal). **Ruichen Wu:** methodology (equal), resources (equal). **Yi Yang:** conceptualization (equal), resources (equal), supervision (equal), writing – review and editing (equal).

## Ethics Statement

All experiments and animal handling were conducted according to research protocols approved by the Animal Welfare and Ethics Committee of Xinjiang Agricultural University (2021079).

## Conflicts of Interest

The authors declare no conflicts of interest.

## Data Availability

The data supporting the findings of this study are publicly available from the National Center for Biotechnology Information (NCBI) Sequence Read Archive (SRA) at https://www.ncbi.nlm.nih.gov/sra/PRJNA1133215, reference number [PRJNA1133215].
