# Correction to “A Comparison of Digestive Strategies for *Teratoscincus roborowskii* With Different Diet Compositions: Digestive Enzyme Activities, Gut Microbiota, and Metabolites”Wang Z, Wu R, Yang Y. A Comparison of Digestive Strategies for *Teratoscincus roborowskii* With Different Diet Compositions: Digestive Enzyme Activities, Gut Microbiota, and Metabolites. Ecology and Evolution, 2024, 14(12): e70751.

**DOI:** 10.1002/ece3.70843

**Published:** 2025-01-08

Wang Z , Wu R , Yang Y . A Comparison of Digestive Strategies for *Teratoscincus roborowskii* With Different Diet Compositions: Digestive Enzyme Activities, Gut Microbiota, and Metabolites. Ecology and Evolution, 2024, 14(12): e70751.39717646 10.1002/ece3.70751PMC11663733

Figure 1, cited in the introduction, and the associated legend is replaced as seen below.

**FIGURE 1:**
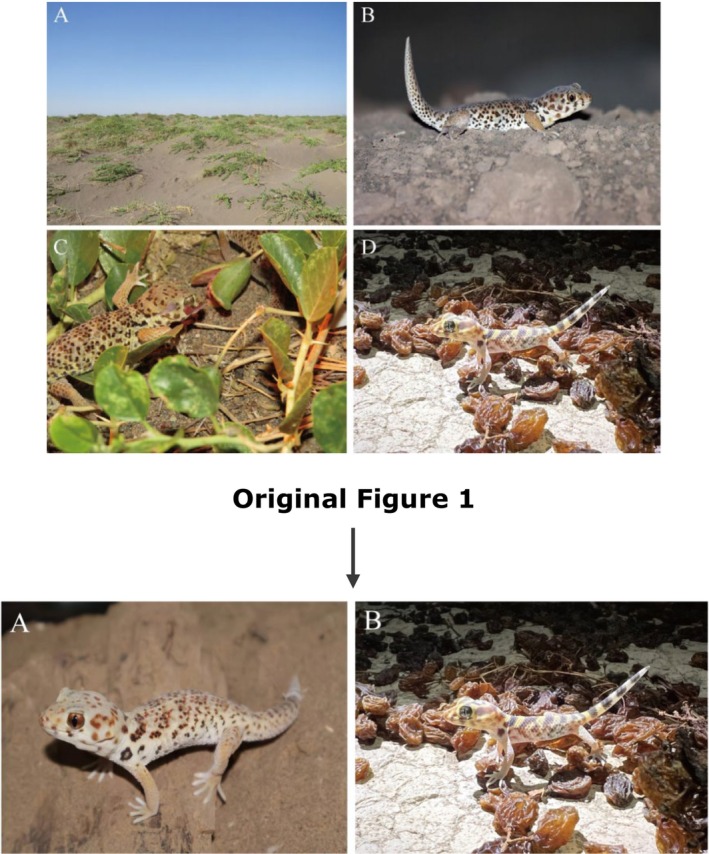
(A) *T.roborowskii*; (B) *T.roborowskii* active in the area where grapes are being dried.

We apologize for this error.

